# Cognitive impairment after a stroke in young adults: A systematic review and meta-analysis

**DOI:** 10.1177/17474930231159267

**Published:** 2023-03-07

**Authors:** Rosemarije PC Weterings, Roy PC Kessels, Frank-Erik de Leeuw, Vitória Piai

**Affiliations:** 1 Donders Institute for Brain, Cognition and Behaviour, Radboud University, Nijmegen, The Netherlands; 2 Department of Neurology, Radboud University Medical Center, Nijmegen, The Netherlands; 3 Department of Medical Psychology, Radboud University Medical Center and Donders Institute for Brain, Cognition and Behaviour, Nijmegen, The Netherlands; 4 Vincent van Gogh Institute for Psychiatry, Venray, The Netherlands

**Keywords:** Aphasia, cognition, language impairment, young-stroke

## Abstract

**Background:**

Information about cognitive functioning is vital in the management of stroke, but the literature is mostly based on data from individuals older than 50 years of age who make up the majority of the stroke population. As cognitive functioning is subject to change due to aging, it is unclear whether such cognitive impairment patterns from the general stroke literature apply to the growing population of younger people with a stroke.

**Aim:**

The aim of the study was to conduct a systematic review and meta-analysis of the proportion and severity of cognitive impairment in young-stroke patients.

**Summary of review:**

MEDLINE, Embase, PsycINFO, and Web of Science were systematically searched up to 11 October 2022. Studies were included if they reported on a population of young-stroke patients, evaluated cognitive functioning as an outcome measure, and reported original data. We estimated the pooled prevalence rates for cognitive impairment and for aphasia. In addition, we calculated the pooled estimates for the severity of impairment per cognitive domain in the chronic phase (defined as >6 months post-stroke). Six hundred thirty-five articles were identified, of which 29 were eligible for inclusion. The pooled prevalence of cognitive impairment was 44% (*k* = 10; 95% confidence interval (CI): 34–54%) and of aphasia 22% (*k* = 13; 95% CI: 12–39%). Young-stroke patients in the chronic phase performed worse than stroke-free healthy age-appropriate controls across all cognitive domains examined, with Hedges’ g effect sizes ranging from −0.49 to −1.64.

**Conclusion:**

Around half of all young-stroke patients present with cognitive impairment and around a quarter with aphasia. Our data suggest that patterns of impairment in young-stroke patients follow those in the general stroke literature.

## Introduction

Post-stroke cognitive impairment is commonly found in many patients.^1,2^ However, the existing literature is mainly based on individuals over 50 years. Especially the incidence of individuals who had a first-ever stroke at a relatively young age (<50 years, young-stroke patients) has significantly increased over the past decade,^3,4^ accounting for approximately 10% of all strokes.^5,6^ Given that aging affects both the brain and cognitive (dys)function after stroke,^7^ it is unknown whether patterns of impairment from the general literature on stroke generalize to young-stroke patients.

Information about post-stroke cognitive functioning (e.g. likelihood and severity of impairment) is essential for young-stroke patients who are often at cross-roads in their lives (planning a family and career moves), as they are confronted with post-stroke sequelae that may affect their lives for the decades to come. Studies on cognition in young-stroke patients are, to date, scarce. Characteristics, study design, and outcomes vary greatly in studies that do report on young-stroke patients. Furthermore, systematic reviews and meta-analyses on post-stroke cognitive impairment in this young population are lacking.

This study aimed to obtain a comprehensive overview of the literature on post-stroke cognitive functioning in young-stroke patients through a systematic review and meta-analyses. We first investigated what measurement tools are used to evaluate cognitive functioning. We then investigated the proportion of reported cognitive impairment (i.e. dichotomous: impairment yes/no) in all stages post-stroke. Given that the field makes a distinction between cognitive impairment and aphasia (a language disorder), we also investigated the proportion of aphasia in this population in all stages post-stroke. In addition, we took a first step in specifying the reported severity of impairment (i.e. quantifying their effect size) per cognitive domain in the chronic phase (>6 months post-stroke onset).

## Methods

We followed the Preferred Reporting Items for Systematic Reviews and Meta-Analyses (PRISMA) guidelines.^8^ Data and code are shared via https://osf.io/u236c/?view_only=2acb53b4ed0149da9d22d45281ce030c.

### Search strategy

We used the following four electronic databases: MEDLINE, Embase, PsycINFO, and Web of Science (Supplementary Data S1). The search strategy was developed with the help of experienced librarians of Radboud University’s library. Briefly, search terms (applied to title, abstract, and keywords) were divided into three groups: stroke (“cerebral vascular accident” or “cerebral hemorrhage” or “ischemia” or “brain infarct,” and related terms), cognition (“cognitive impairment” or “cognitive dysfunction” or “neuropsychological deficit” or “neuropsychological assessment,” and related terms), and language (“linguistic” or “aphasia” or “communication” or “word fluency,” and related terms). We included a specific term for language, as this concept is not always placed under the term *cognition*. The results of this search were then restricted to samples <50 years of age (details in Supplementary Data S1). The search was carried out on 23 December 2021 (updated on 11 October 2022). The search results were exported to Covidence.^9^ Two independent reviewers screened titles and abstracts, and full texts. If there was disagreement at any phase, consensus was reached by discussion.

### Eligibility criteria and study selection

Inclusion criteria were (1) young-adult population (18–55 years at onset) with a clinical diagnosis of stroke, (2) cognitive functioning evaluated as outcome measure, and (3) reporting original data. We placed restrictions neither on study design (i.e. qualitative studies, randomized controlled trials, and observational studies) nor on phase post-onset, as long as studies were peer-reviewed. Conference summaries/abstracts, reviews, and case studies were excluded. There were no restrictions on the language the article was written in. If multiple articles reported results of the same task(s) for the same cohort, we included the article with the largest sample size and the most relevant details to avoid duplicated data. If different articles studying the same cohort reported different outcome measures, we included both articles.

Specifically for the severity of impairment meta-analysis, we selected only articles from which an effect size could be calculated. In addition, we restricted the severity of impairment meta-analysis to articles reporting on the chronic phase after stroke (>6 months post-onset) to decrease heterogeneity and increase the clinical relevance of our findings.

### Quality assessment

We based our quality assessment on Sexton et al.’s^10^ version of the Crowe Critical Appraisal Tool.^11^ Our criteria evaluated the quality and suitability of the included studies’ participant selection, data collection, and outcomes (Supplementary Data S2). An overall quality score was calculated (maximum 9 points).

### Data extraction

Data were extracted using a standard form developed for our study and checked by a research assistant. Extracted data per study included number of people with a stroke, population characteristics (country, sex, and age), stroke characteristics (type of stroke, severity at presentation, and time since stroke at testing), study restrictions (reporting only first-ever stroke, exclusion of people with aphasia or dementia), and outcome measurement (assessment method, type of neuropsychological tests, and definition of impairment).

For the meta-analyses on the proportion of impairment, we extracted the number of people with/without impairment. Given that the literature categorizes language impairment separately (i.e. aphasia) from cognitive impairment, the analyses were separated for cognitive and language impairment.

For the meta-analyses on severity of impairment, we extracted the respective performance of patients on neuropsychological tests and of stroke-free healthy age-appropriate controls. If a study did not provide the performance of a control group, we searched for normative data of the test, matched, where possible, for age, sex, and education.

#### Effect size estimation

For calculating the severity of impairment in different cognitive domains, we distinguished between the cognitive domains following Lezak et al.’s^12^ classification: global cognition, visuoconstruction, language, attention and executive functioning, delayed memory, immediate memory, working memory, and processing speed (note that these domains should not be interpreted too strictly). Then, per study and cognitive domain, we estimated Hedges’ g, a measure of effect size corrected for small sample sizes, and its corresponding estimated sampling variance (sample-size-averaged estimator)^13^ using the R^14^ (version 4.1.2) package esc^15^ in the following way: per task, we took the reported mean and standard deviation (SD) to calculate Hedges’ g. For scores reported with a median and interquartile range, we first calculated the mean and SD using the given median and range.^16^ When multiple tasks were used for the same cognitive domain in one study, we calculated z-scores for the patient group per task (mean patient – mean controls / SD controls). We then took the mean and SD of the z-scores within that domain for calculating Hedges’ g per cognitive domain per study.

### Meta-analyses

Inferential statistical analyses were carried out with R^14^ (package metafor).^17^ The pooled prevalence rates for impairment were assessed with random-effects meta-analysis for binominal distributions, using 95% confidence intervals (CIs) (function “metaprop”), based on the study’s stroke-sample size (number of observations) and the number of people with an impairment (number of events).

The pooled estimates for the severity of impairment per domain were assessed with random-effects meta-analysis when there was a sufficient number of studies (*k* ⩾ 5) reporting on a specific domain, and otherwise with fixed-effects models (minimum *k* = 2, otherwise the cognitive domain was not analyzed).^18^ We set the alpha level at 0.05 for the severity meta-analyses. We considered effect sizes between 0.2 and 0.5 as small, between 0.5 and 0.8 as medium, and >0.8 as large.^19^

We quantified and evaluated heterogeneity in the meta-analyses by the *I*^2^ statistics and by visually checking the forest plots with the overlap of the CIs. We considered the level of heterogeneity based on the guidelines of the Cochrane Handbook,^20^ with values over 75% representing considerable heterogeneity.

## Results

### Included articles

**Figure 1.**
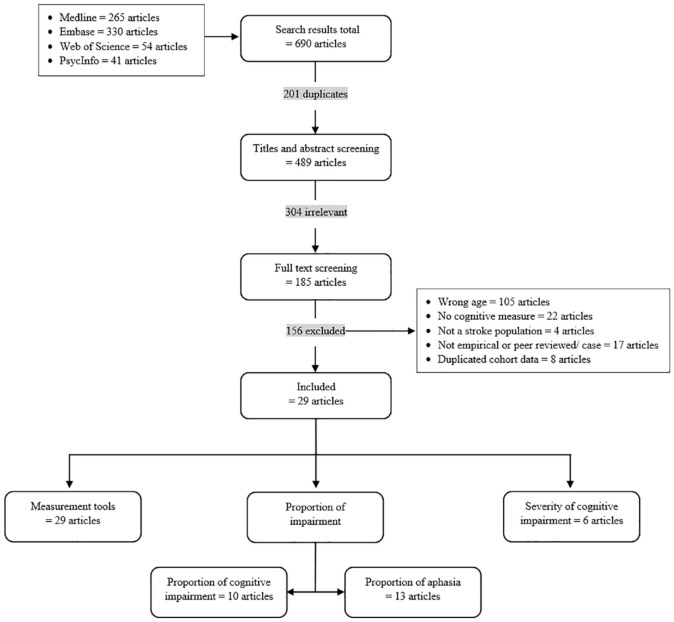
Flowchart study selection.

Six hundred ninety articles were identified. After removing duplicates (201) and irrelevant articles (460), 29 articles were eligible for inclusion (Figure 1). It occurred twice that two articles reported on the same cohort (Hordaland County cohort Norway^21,22^ and Sahlgrenska Academy Study on Ischemic Stroke Sweden),^23,24^ but provided different information; thus, we included both. More than half of the studies (16/29) included a population of ischemic stroke only^22–37^ (Table 1). Time since stroke while evaluating cognitive outcomes ranged from a few hours to 11 years, but almost half of the studies (13/29) reported on people in the chronic phase^21–24,26,28,30,31,36,38–41^ and the other half (14/29) included people in the non-chronic phase.^25,27,29,32–35,37,42–47^ Two studies included both the chronic and non-chronic phase.^48,49^ The studies were conducted in 22 different countries. Exclusion criteria related to the ability to participate were common across studies: 11 studies reported to have excluded people with aphasia^23,26,28,30,32,35,36,43,45–47^ and 6 studies people with dementia.^23,32,40,41,45,47^

**Table 1.** Characteristic of the included studies (*k* = 29).

Study	Country	Population characteristics		Stroke characteristics				Restrictions	
		N (men%)	Age in years	Type of stroke	Time since stroke at testing	Period	Hemisphere (n or %)	First-ever stroke	Excl
Aarnio et al.^25^	FIN	769 (62%)	M = 44.0	IS	At hospital discharge	Chronic	Nr.	Yes	—
Cao et al.^26^	ITA	40 (40%)	M = 38.8 (SD = 8.3)	IS	9.2 m	Non-chronic	L: n = 15 R: n = 18	Yes	A
Chraa et al.^27^	MAR	128 (59%)	M = 28.3 (SD = 4.2)	IS	At clinical presentation	Non-chronic	Nr.	Nr.	—
de Bruijn et al.^28^	NLD	96 (46%)	Md = 43.0	IS	5 y	Chronic	L: n = 47 R: n = 42 L + R: n = 7	Yes	A
Dieynabou Sow et al.^42^	SEN	53 (43%)	M = 42.1	ICH	16 h	Non-chronic	L: 60%	No	—
Do et al.^37^	TWN	6512 (65%)	M = 37.2 (SD = 6.1)	IS	During first stroke admission	Non-chronic	Nr.	Yes	—
Done et al.^43^	IND	150 (73%)	M = 39.3 (SD = 6.6)	IS ICH	24 m	Chronic	Nr.	Nr.	A
Ferro and Crespo^48^	PRT	254 (47%)	<40 y: n = 102 41–50 y: n = 152	Stroke	0–1 m: n = 142 2–3 m: n = 36 >6 m: n = 54	Non-chronic & chronic	L: n = 234 R: n = 20	Nr.	—
Gans et al.^49^	USA	121 (57%)	M = 39.8 (SD = 8.2)	IS TIA ICH	0–3 m: n = 97 3–12 m: n = 89	Non-chronic & chronic	Nr.	Yes	—
Gonzalez Mc et al.^29^	CHL	31 (Nr.)	0–24 y: n = 2 25–34 y: n = 2 35–44 y: n = 7 45–54 y: n = 20	IS	At clinical presentation	Non-chronic	Nr.	Yes	—
Hoffmann^44^	ZAF	172 (49%)	M = 43.8	Stroke	1–4 w	Non-chronic	Nr.	Yes	—
Hoffmann and Cases^45^	USA	26 (39%)	Nr.	ST PO	1 m	Non-chronic	L: n = 9 R: n = 14 L + R: n = 3	Nr.	A D
Huang et al.^30^	CHN	350 (68%)	M = 41.0 (SD = 6.8)	IS	5.8 y	Chronic	L: n = 152 R: n = 148 p: n = 50	Yes	A
Kapoor et al.^46^	CAN	57 (39%)	M = 40.9 (SD = 8.1)	IS TIA	55 d	Non-chronic	Nr.	No	A
Kim et al.^31^	KOR	96 (80%)	M = 39 (SD = 7)	IS	1–5 y	Chronic	L: n = 45 R: n = 41 L + R: n = 10	Yes	—
Koivunen et al.^38^	FIN	76 (53%)	Nr.	ICH	9.7 y	Chronic	Nr.	Yes	—
Lu et al.^32^	CHN	84 (58%)	M = 43.5 (SD = 5.8)	IS	14 d	Non-chronic	Nr.	Yes	A D
Lutski et al.^33^	ISR	336 (63%)	Nr.	IS	Hospitalization period	Non-chronic	Nr.	Yes	—
Mattuzzi and Pfenninger^39^	divers	26 (Nr.)	17–25 y: n = 11 26–30 y: n = 14 40–50 y: n = 1	Stroke	Chronic	Chronic	Nr.	Nr.	—
Moond et al.^34^	IND	160 (74%)	M = 36.2	IS	At clinical presentation	Non-chronic	Nr.	No	—
Naess et al.^21^	NOR	193 (Nr.)	Nr.	Stroke	6.0 y	Chronic	Nr.	Yes	—
Naess et al.^22^	NOR	195 (58%)	M = 42	IS	6.0 y	Chronic	Nr.	Yes	—
Pedersen et al.^23^	SWE	67 (48%)	M = 40 (SD = 8)	IS	7 y	Chronic	Nr.	No	A D
Pinter et al.^35^	AUT	114 (64%)	M = 45.5 (SD = 9.5)	IS	6 d	Non-chronic	L: 45% R: 39%	Nr.	A
Rebchuk et al.^47^	CAN	52 (37%)	Md = 47.0 (IQR = 38.5–51.0)	IS TIA ICH	90 d	Non-chronic	L: n = 19 R: n = 16 L + R: n = 1 c/b: n = 11 m: n = 3 u = 2	Yes	A D
Samuelsson et al.^24^	SWE	142 (57%)	M = 43 (SD = 9.3)	IS	7 y	Chronic	L: n = 58 R: n = 55 c/b: n = 22 m: n = 7	No	—
Saroja et al.^40^	IND	92 (59%)	<20 y: n = 5 21-30 y: n = 37 31-40 y: n = 30 41-50 y: n = 20	CVST	2.2 y	Chronic	Nr.	Nr.	D
Schaapsmeerders et al.^36^	NLD	277 (44%)	M = 40 (SD = 7.7)	IS	11.0 y	Chronic	L: n = 16 R: n = 102 L + R: n = 7	Yes	A
Si Larbi et al.^41^	SAU	710 (68%)	M = 44.54 (SD = 9.27)	Stroke	6 m	Chronic	Nr.	No	D

Excl: excluded; Country: abbreviation is country code; M: mean; IS: ischemic stroke; chronic: >6 months post-stroke; Nr.: not reported; SD: standard deviation; non-chronic: 0–6 months post-stroke; N: number of people; A: people with aphasia; D: people with dementia; Md: median; ICH: intracerebral hemorrhage; h: hours; d: days; w: weeks; m: months; y: years; p: posterior; c/b: cerebellum and/or brainstem; m: mixed; u: unknown; TIA: transient ischemic attack; ST: isolated cerebellar or brainstem subtentorial stroke; PO: parieto-occipital lobe infarct; IQR: interquartile range; CVST: cerebral venous sinus thrombosis.

### Study quality

The mean quality score of the 29 studies was 6.59 (out of 9 points, SD = 1.49; range = 4–9; median = 6.5, Supplementary Table S1). All studies included at least some information about participant and stroke characteristics. Stroke type was mostly reported (24/29).^22–38,40,42,43,45–47,49^ Time since stroke was available in all studies. Sixteen studies reported that they included only first-ever stroke.^21,22,25,26,28–33,36–38,44,47,49^ Lesion side (left or right hemisphere) was reported in 10 studies.^24,26,28,30,31,35,36,42,45,47^ Stroke was specified with a definition in almost all studies (24/29).^21–23,25–32,34–38,40–42,44–48^ Studies scored the lowest on operationalization of outcome measures and criteria (e.g. only 13 studies^21–23,25,26,28,30,32,35,36,38,43,47^ operationalized impairment in detail with specific cut-off criteria on the administered tests). Appropriateness of the manner of assessment also varied between studies. Ten studies^27,29,31,33,34,37,39,41,42,49^ used a subjective test or did not report any tests results, seven studies^21,25,30,38,40,46,47^ used a cognitive test that only provided a global cognitive score, and 12 studies^22–24,26,28,32,35,36,43–45,48^ used tests tapping into specific cognitive domains. All reported in detail how they collected data.

### Measurement tools

All 29 studies could be used to describe what measurement tools are used to evaluate cognitive functioning after stroke in young adults (Supplementary Table S2). Fourteen studies used a test that measured global cognition (e.g. the Mini-mental state examination).^21,23,24,26,30,32,35,36,38,40,43,44,46,47^ Twelve studies used domain-specific neuropsychological tests.^22–26,28,32,35,36,44,45,48^ Seven studies reported using both a global cognition and domain-specific tests.^23,24,26,32,35,36,44^ Five studies used self-report questionnaires.^24,31,39,42,49^ Six studies did not report on the use of any test, but still reported on cognitive functioning.^27,29,33,34,37,41^

### Proportion of impairment

**Figure 2.**
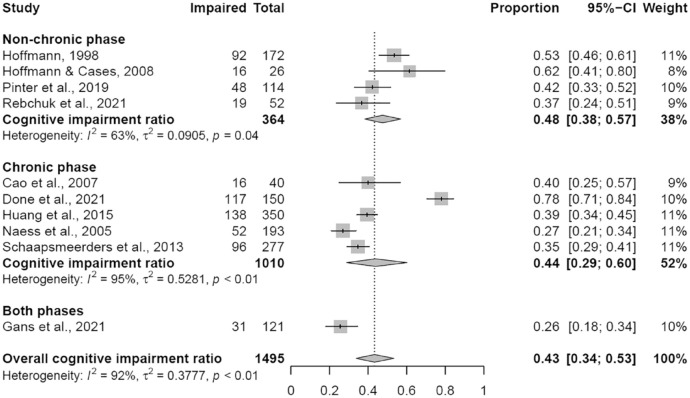
Forest plot for the proportion of cognitive impairment. Non-chronic phase: 0–6 months post-stroke; chronic phase: >6 months post-stroke.

Ten of the 29 studies were eligible for determining the proportion of cognitive impairment other than aphasia (total N = 1495, Supplementary Table S3).^21,26,30,35,36,43–45,47,49^ Eight of these 10 studies quantified impairment as their outcome measure by providing a cut-off score on a test. Seven of these 10 studies excluded people with aphasia.^26,30,35,36,43,45,47^ Overall, the pooled prevalence was 44% (*k* = 10, 95% CI: 34–54%, Figure 2). However, heterogeneity was very high (*I*^2^ = 92%, *p* < 0.01). For the two different phases after stroke analyzed separately, the pooled prevalence in the non-chronic phase was 48% (based on *k* = 4, N = 364; 95% CI: 38–57%, *I*^2^ = 63%, *p* = 0.04) and in the chronic phase 44% (based on *k* = 5, N = 1010; 95% CI: 29–60%, *I*^2^ = 95%, *p* < 0.01).

**Figure 3.**
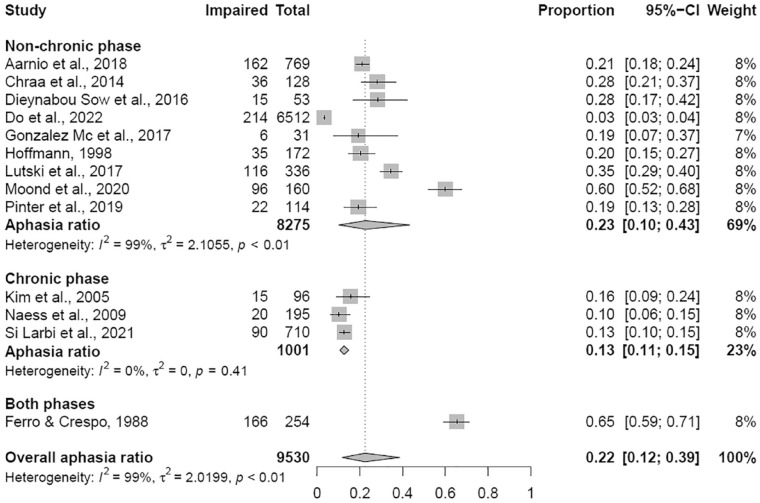
Forest plot for the proportion of aphasia. Non-chronic phase: 0–6 months post-stroke; chronic phase: >6 months post-stroke.

Thirteen of the 29 studies were eligible for determining the proportion of aphasia (total: N = 9530, Supplementary Table S4).^22,25,27,29,31,33–35,37,41,42,44,48^ Five of these 13 studies quantified aphasia as their outcome measure by providing a cut-off score on a language test. The pooled prevalence was 22% (*k* = 13, 95% CI: 12–39%, Figure 3). However, heterogeneity was very high (*I*^2^ = 99%, *p* < 0.01). For the two different phases after stroke analyzed separately, the pooled prevalence in the non-chronic phase was 23% (based on *k* = 9; N = 8275; 95% CI: 10–43%, *I*^2^ = 99%, *p* < 0.01) and in the chronic phase 13% and not statistically significant (based on *k* = 3; N = 1001; 95% CI: 11–15%, *I*^2^ = 0%, *p* = 0.41).

### Severity of impairment per domain

**Figure 4.**
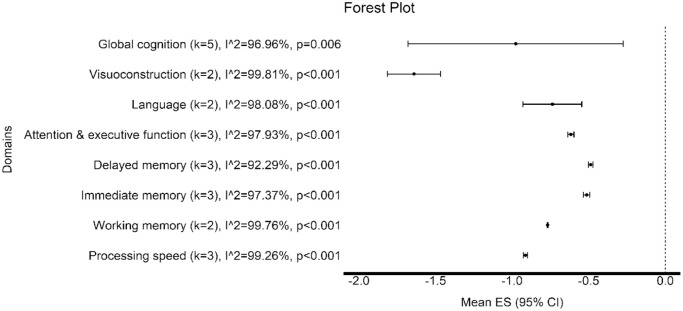
Summary of the meta-analyses for the severity of impairment per cognitive domain in the chronic phase (>6 months post-stroke). *k* = total number of studies; worse performance by the young-stroke patients compared to stroke-free healthy age-appropriate controls is indicated by a negative sign of the effect sizes. Cognitive domains are based on Lezak et al.^12^

Six of the 29 studies were eligible for the meta-analysis on severity of the impairment per domain (Supplementary Table S5).^21,23,26,28,36,38^ Overall, young-stroke patients performed worse than stroke-free healthy age-appropriate adults across all cognitive domains (all Hedges’ gs > –0.487, all ps ⩽ 0.006, Figure 4, Supplementary Figures S1–S8), with effect sizes ranging from small (delayed memory), medium (attention and executive functioning, immediate memory, language, and working memory) to large (global cognition, visuoconstruction, and processing speed).

## Discussion

Our results showed that almost half of the young adults had a cognitive impairment after stroke (often after excluding people with aphasia from the sample) and around a quarter had aphasia. When inspecting the non-chronic and chronic phases separately, particularly the proportion of aphasia was smaller in the chronic phase than in the non-chronic phase, which is also known from the general aphasia literature.^50^ Note, however, that these analyses were cross-sectional rather than longitudinal. Given that we could not analyze the data as a function of time post-onset, it is less straightforward to relate these numbers to prevalence numbers in the literature.^51–53^

By contrast, we found that the prevalence of cognitive impairment in the chronic phase is relatively similar to the non-chronic phase. When inspecting the studies in the chronic phase, there is one outlier^43^ with a high prevalence of cognitive impairment, which could drive the height of the prevalence in the chronic phase. However, since the quality score of this study is the same as the average, we did not see a reason for exclusion. When zooming in on the severity of the impairment in different cognitive domains in the chronic phase, our results showed that young-stroke patients performed significantly worse on all domains than stroke-free healthy age-appropriate controls, with small to large effect sizes.

This systematic review further showed that studies evaluating cognitive function in young-stroke patients are scarce, and the comprehensiveness of the testing is low. In the studies that did investigate cognitive functioning in young-stroke patients, a variety of measurement tools was used (as often happens in the general stroke literature).^54^ In addition, clinical classifications (impaired or not) are not always based on well-described quantitative criteria.

For this review, we took an inclusive approach, which is a strength of this study. We included studies of high and lower quality, all types of stroke, studies from many (also non-western) countries, and articles written in different languages, making our findings more generalizable to a broader population of young-stroke patients. The downside of this approach is the inevitable variability between the studies examining cognition in young-stroke patients. Hence, our conclusions are limited by the quality of the literature included.

Studies examined different time phases post-stroke (non-chronic or chronic), for which it is known that severity of impairment differs.^53^ In the stroke literature, there is, in general, a lack of consensus how cognitive impairment is defined following test results and the type of tests that are used,^54^ which we also encountered in this review. Another limitation of the literature so far may be the exclusion of participants that are seen as ineligible for performing cognitive tests either by their aphasia or stroke severity.^55^ In our review, we found that about two-fifths of the studies that investigated cognitive functioning excluded people with aphasia. It is easier to exclude those patients because of potential problems with understanding the tests. However, this yields a skewed picture of the young-stroke patient population. These factors together could affect estimations of prevalence and severity of the impairments. While these are not limitations by the studies themselves, it does impact the conclusions we could draw from them. The high heterogeneity we found could be an indication of this issue and our study wished, therefore, to remain cautious in drawing very strong conclusions.

Our results confirm the presence of cognitive impairment after stroke in young adults and give a first indication that the pattern seems to align with the general stroke literature, which is based mostly on older individuals. Clinicians could use our results to better inform their young-adult patients about their prognosis after stroke. Moreover, a clearer picture of the cognitive profile will help establish targets for neurorehabilitation. Nevertheless, a relevant question for clinical practice that remains unanswered is how this cognitive profile predicts functional outcomes. Future studies should consider collecting functional outcome measures (e.g. return to work) and report the results on cognitive functioning in the stroke population by different age groups, together with providing an explicit quantifiable definition of impairment.

## Supplemental Material

sj-docx-1-wso-10.1177_17474930231159267 – Supplemental material for Cognitive impairment after a stroke in young adults: A systematic review and meta-analysis Click here for additional data file. Supplemental material, sj-docx-1-wso-10.1177_17474930231159267 for Cognitive impairment after a stroke in young adults: A systematic review and meta-analysis by Rosemarije PC Weterings, Roy PC Kessels, Frank-Erik de Leeuw and Vitória Piai in International Journal of Stroke
