# Supplementary material for: Cognitive impairment after a stroke in young adults: A systematic review and meta-analysis
Source: Int J Stroke. 2023 Mar 7;18(8):888–97. doi: 10.1177/17474930231159267 (PMC10507997; doi:10.1177/17474930231159267)
Supplement: sj-docx-1-wso-10.1177_17474930231159267 – Supplemental material for Cognitive impairment after a stroke in young adults: A systematic review and meta-analysis [file sj-docx-1-wso-10.1177_17474930231159267.docx]

**Supplementary material**

**Cognitive and language impairment after a stroke in young adults: a systematic review and meta-analysis**

Rosemarije P.C. Weterings, Roy P.C. Kessels, Frank-Erik de Leeuw, Vitória Piai

**Supplementary Data**

Supplementary Data S1: Search strategies per data base

Supplementary Data S2: Quality Appraisal Criteria

**Supplementary Tables**

Supplementary Table S1.Quality assessment of the included studies (*k* = 29)

Supplementary Table S2. Overview of the used cognitive measurement tools

Supplementary Table S3. Summary of the studies (*k* = 10) reporting the prevalence of cognitive impairment in young stroke

Supplementary Table S4. Summary of the studies (*k* = 13) reporting the prevalence of language impairment in young stroke

Supplementary Table S5. Summary of the studies (*k* = 6) reporting the severity of impairment for different cognitive domains

**Supplementary Figures**

Supplementary Figure S1. Forest plot of the severity of impairment in global cognition

Supplementary Figure S2. Forest plot of the severity of impairment in visuoconstruction

Supplementary Figure S3 Forest plot of the severity of impairment in language

Supplementary Figure S4. Forest plot of the severity of impairment in attention and executive functioning

Supplementary Figure S5. Forest plot of the severity of impairment in delayed memory

Supplementary Figure S6. Forest plot of the severity of impairment in immediate memory

Supplementary Figure S7. Forest plot of the severity of impairment in working memory

Supplementary Figure S8. Forest plot of the severity of impairment in processing speed

**Reference list** for supplementary material

**Supplementary data**

**Data S1: Search strategies per data base**

*****In blue, search update on 11^th^ October 2022

*S1.1. Search strategy Medline*

Ovid MEDLINE(R) ALL <1946 to December 21, 2021>

1        Neuropsychological findings in young-adult stroke patients.m_titl.        1

2        Long-Term Cognitive Outcome of Ischaemic Stroke in Young Adults.m_titl.        1

3        Dynamic Trajectory of Long-Term Cognitive Improvement Up to 10 Years in Young Community-Dwelling Stroke Survivors: A Cohort Study.m_titl.        1

4        "Subjective cognitive failures after stroke in young adults: Prevalent but not related to cognitive impairment".m_titl.        1

5        Aphasia among Young Patients with Ischemic Stroke on Long-term Follow-up.m_titl.        1

6        (Prevalence and short-term changes of cognitive dysfunction in young ischaemic stroke patients).m_titl.        1

7        Long-term cognitive impairment after first-ever ischemic stroke in young adults.m_titl.        1

8        1 or 2 or 3 or 4 or 5 or 6 or 7        7

9        (cerebrovascular accident* or cerebral vascular accident* or cerebrovascular disorder* or cerebral vascular disorder* or cerebrovascular infarct* or cerebral vascular infarct* or cerebral hemorrhage or stroke or ischemia or brain infarct*).ti,ab,kf,hw.        625779

10        (cognition or cognitive impairment* or cognitive dysfunction* or cognitive function* or cognitive condition* or cognitive defect* or cognitive complaint*).ti,ab,kf,hw.        307715

11        (neuropsychology or neuropsychological deficit* or neuropsychological assessment* or neuropsychological investigation*).ti,ab,kf,hw.        17158

12        (language* or linguistic* or aphasia or verbal communication or oral communication or speech or word fluency).ti,ab,kf,hw.        326874

13        10 or 11 or 12        616080

14        9 and 13        25316

15        (young adj5 stroke).ti,ab,kf,hw.        4157

16        14 and 15        315

17        limit 14 to ("young adult (19 to 24 years)" or "adult (19 to 44 years)" or "young adult and adult (19-24 and 19-44)" or "middle age (45 to 64 years)")        10761

18        8 and 16        7

19        limit 16 to case reports        69

20        16 not 19        246

21 limit 20 to yr="2022" 19*

*S1.2. Search strategy Embase*

Embase <1974 to 2021 December 21>

1        Neuropsychological findings in young-adult stroke patients.m_titl.        1

2        Long-Term Cognitive Outcome of Ischaemic Stroke in Young Adults.m_titl.        3

3        Dynamic Trajectory of Long-Term Cognitive Improvement Up to 10 Years in Young Community-Dwelling Stroke Survivors: A Cohort Study.m_titl.        1

4        "Subjective cognitive failures after stroke in young adults: Prevalent but not related to cognitive impairment".m_titl.        1

5        Aphasia among Young Patients with Ischemic Stroke on Long-term Follow-up.m_titl.        1

6        (Prevalence and short-term changes of cognitive dysfunction in young ischaemic stroke patients).m_titl.        1

7        Long-term cognitive impairment after first-ever ischemic stroke in young adults.m_titl.        2

8        1 or 2 or 3 or 4 or 5 or 6 or 7        10

9        (cerebrovascular accident* or cerebral vascular accident* or cerebrovascular disorder* or cerebral vascular disorder* or cerebrovascular infarct* or cerebral vascular infarct* or cerebral hemorrhage or stroke or ischemia or brain infarct*).ti,ab,kf,hw.        965370

10        (cognition or cognitive impairment* or cognitive dysfunction* or cognitive function* or cognitive condition* or cognitive defect* or cognitive complaint*).ti,ab,kf,hw.        520877

11        (neuropsychology or neuropsychological deficit* or neuropsychological assessment* or neuropsychological investigation*).ti,ab,kf,hw.        39941

12        (language* or linguistic* or aphasia or verbal communication or oral communication or speech or word fluency).ti,ab,kf,hw.        446730

13        10 or 11 or 12        934907

14        9 and 13        55545

15        (young adj5 stroke).ti,ab,kf,hw.        5355

16        14 and 15        539

17        limit 14 to adult <18 to 64 years>        21015

18        8 and 16        10

19        limit 16 to conference abstract status        237

20        16 not 19        302

 21 limit 20 to yr="2022" 28*

*S1.3. Search strategy PsycInfo*

APA PsycInfo <1806 to December Week 2 2021>

1        Neuropsychological findings in young-adult stroke patients.m_titl.        1

2        Long-Term Cognitive Outcome of Ischaemic Stroke in Young Adults.m_titl.        0

3        Dynamic Trajectory of Long-Term Cognitive Improvement Up to 10 Years in Young Community-Dwelling Stroke Survivors: A Cohort Study.m_titl.        0

4        "Subjective cognitive failures after stroke in young adults: Prevalent but not related to cognitive impairment".m_titl.        0

5        Aphasia among Young Patients with Ischemic Stroke on Long-term Follow-up.m_titl.        0

6        (Prevalence and short-term changes of cognitive dysfunction in young ischaemic stroke patients).m_titl.        1

7        Long-term cognitive impairment after first-ever ischemic stroke in young adults.m_titl.        0

8        1 or 2 or 3 or 4 or 5 or 6 or 7        2

9        (cerebrovascular accident* or cerebral vascular accident* or cerebrovascular disorder* or cerebral vascular disorder* or cerebrovascular infarct* or cerebral vascular infarct* or cerebral hemorrhage or stroke or ischemia or brain infarct*).ti,ab,id,hw.        47054

10        (cognition or cognitive impairment* or cognitive dysfunction* or cognitive function* or cognitive condition* or cognitive defect* or cognitive complaint*).ti,ab,id,hw.        195856

11        (neuropsychology or neuropsychological deficit* or neuropsychological assessment* or neuropsychological investigation*).ti,ab,id,hw.        42239

12        (language* or linguistic* or aphasia or verbal communication or oral communication or speech or word fluency).ti,ab,id,hw.        341489

13        10 or 11 or 12        542804

14        9 and 13        9776

15        (young adj5 stroke).ti,ab,id,hw.        418

16        14 and 15        48

17        8 and 16        2

18        limit 14 to ("300 adulthood <age 18 yrs and older>" or 320 young adulthood <age 18 to 29 yrs> or 340 thirties <age 30 to 39 yrs> or 360 middle age <age 40 to 64 yrs>)        6420

19        limit 16 to "0200 clinical case study"        11

20        16 not 19        37

21 limit 20 to yr="2022" 4*

A4. Search strategy Web of Science

*S1.4. Web of Science*

TS=(("cerebrovascular accident*" or "cerebral vascular accident*" or "cerebrovascular disorder*" or "cerebral vascular disorder*" or "cerebrovascular infarct*" or "cerebral vascular infarct*" or "cerebral hemorrhage" or "stroke" or "ischemia" or "brain infarct*") AND (cognition or "cognitive impairment*" or "cognitive dysfunction*" or "cognitive function*" or "cognitive condition*" or "cognitive defect*" or "cognitive complaint*" OR "neuropsychology" or "neuropsychological deficit*" or "neuropsychological assessment*" or "neuropsychological investigation*" OR "language*" or "linguistic*" or "aphasia" or "verbal communication" or "oral communication" or "speech" or "word fluency") AND ("young stroke")) 50 and 2022 (Publication Years) 4*

**Supplementary Data S2: Quality Appraisal Criteria**

Based on Sexton et al. (2019) version of the Crowe Critical Appraisal Tool (Crowe & Sheppard, 2011).

Quality of participant selection

**Flow of participants through research**

0 Absent

0.5 Present: Narrative description

1 Present: Flow chart

**Demographic characteristics of participants** (sex, education, nationality)

0 Absent

0.5 Partly present: Mentioned, at least one item mentioned

1 Present: Adequately described, alle present

**Stroke characteristics** (stroke type, hemisphere, time since stoke, first-ever stroke)

0 Absent

0.5 Partly present: Mentioned, at least one item mentioned

1 Present: Adequately described, all present

Quality of the outcome

**Specification of (cognitive/language) impairment criteria**

0 Not specified: No or very little information given

0.5 Less clearly specified: Reference given for criteria used, but no further information (e.g., reliance on clinical judgement without specifying standard criteria used)

1 Clearly specified: Precise information on how operationalized, e.g., number of
 domains of impairment, criteria for functional impairment, and source of normative
 data

**Appropriateness of assessment mode**

0 Not appropriate: Non-validated measure or non-established criteria (i.e., no reference given for assessment mode, no test reported, or uses self-reports/clinical judgements)

0.5 Partially appropriate: Uses a validated, established approach bus uses a global
 cognitive test

1 Most appropriate: Established criteria based on neurocognitive battery measuring specific cognitive domains.

Quality of the data collection

**Data collection method for cognitive data** (specify method for dates, places)

0 Absent

0.5 Partly present: Mentioned

1 Present: Adequately described

**Suitability of data collection for cognitive data** (personnel, materials, processes)

0 Not suitable: Non-standardised tests or untrained assessors, or clinical judgement that doesn't take into account objective criteria

0.5 Partly suitable

1 Most suitable: Trained assessors using standardised testing, if clinical judgement used, must be based on reported objective data and criteria

**Data collection method for stroke ascertainment** (specified method/definition for diagnosis)

0 Absent

0.5 Partly present

1 Present

**Suitability of data collection for stroke case ascertainment**

0 Not suitable: Not clearly specified, self-reported doctor diagnosis

0.5 Less suitable: Clinical diagnosis with not clear reported positive imaging evidence

1 Most suitable: Clinical diagnosis confirmed on positive imaging

**Total = 9**

**Supplementary Tables**

**Supplementary Table S1.** Quality assessment of the included studies (*k* = 29)

| Study (country) | Quality of participant selection | | | Quality of the outcome | | Quality of the data collection | | | | Total score |
| --- | --- | --- | --- | --- | --- | --- | --- | --- | --- | --- |
|  | Participants flow | Demographic characteristics | Stroke characteristics | Specification of impairment criteria | Appropriate-ness of assessment mode | Data collection method (for cognitive data) | Suitability of data collection (for cognitive data) | Data collection method for stroke ascertainment | Suitability of data collection (for stroke case ascertainment) |  |
| Aarnio et al., 2018 | 1 | 0.5 | 0.5 | 1 | 0.5 | 1 | 1 | 1 | 1 | 7.5 |
| Cao et al., 2007 | 0.5 | 1 | 1 | 1 | 1 | 1 | 1 | 1 | 1 | 8.5 |
| Chraa et al., 2014 | 0.5 | 0.5 | 0.5 | 0.5 | 0 | 1 | 1 | 1 | 1 | 6 |
| de Bruijn et al., 2014 | 1 | 1 | 1 | 1 | 1 | 1 | 1 | 1 | 0.5 | 7.5 |
| Dieynabou Sow et al., 2016 | 0.5 | 0.5 | 0.5 | 0.5 | 0 | 1 | 1 | 1 | 1 | 6 |
| Do et al., 2022 | 1 | 0.5 | 0.5 | 0 | 0 | 0,5 | 0 | 1 | 1 | 4.5 |
| Done et al., 2021 | 0.5 | 1 | 0.5 | 1 | 1 | 1 | 1 | 0 | 0 | 6 |
| Ferro & Crespo, 1988 | 0.5 | 0.5 | 0.5 | 0.5 | 1 | 1 | 1 | 1 | 1 | 7 |
| Gans et al., 2021 | 1 | 0.5 | 0.5 | 0.5 | 0 | 1 | 1 | 0 | 0 | 4.5 |
| Gonzalez Mc et al., 2017 | 0.5 | 0.5 | 0.5 | 0 | 0 | 1 | 1 | 1 | 0 | 4.5 |
| Hoffmann, 1998 | 0.5 | 0.5 | 0.5 | 0.5 | 1 | 1 | 1 | 1 | 1 | 7 |
| Hoffmann & Cases, 2008 | 0.5 | 0.5 | 0.5 | 0.5 | 1 | 1 | 1 | 1 | 1 | 7 |
| Huang et al., 2015 | 1 | 1 | 1 | 1 | 0.5 | 1 | 1 | 1 | 1 | 8.5 |
| Kapoor et al., 2019 | 0.5 | 1 | 0.5 | 0 | 0.5 | 1 | 1 | 1 | 0.5 | 6 |
| Kim et al., 2005 | 0.5 | 1 | 1 | 0 | 0 | 1 | 1 | 1 | 1 | 6.5 |
| Koivunen et al., 2015 | 0.5 | 0.5 | 0.5 | 1 | 0.5 | 1 | 1 | 1 | 1 | 7 |
| Lu et al., 2016 | 1 | 1 | 0.5 | 1 | 1 | 1 | 1 | 1 | 1 | 8.5 |
| Lutski et al., 2017 | 1 | 0.5 | 0.5 | 0 | 0 | 1 | 1 | 0 | 0 | 4 |
| Mattuzzi & Pfenninger, 2018 | 0.5 | 1 | 0.5 | 0 | 0 | 1 | 1 | 0 | 0 | 4 |
| Moond et al., 2020 | 0.5 | 0.5 | 0.5 | 0 | 0 | 1 | 1 | 1 | 1 | 5.5 |
| Naess et al., 2005 | 0.5 | 1 | 0.5 | 1 | 0.5 | 1 | 1 | 1 | 1 | 7.5 |
| Naess et al., 2009 | 0.5 | 0.5 | 0.5 | 1 | 1 | 1 | 1 | 1 | 1 | 7.5 |
| Pedersen et al., 2018 | 1 | 1 | 0.5 | 1 | 1 | 1 | 1 | 1 | 0.5 | 8 |
| Pinter et al., 2019 | 1 | 1 | 0.5 | 1 | 1 | 1 | 1 | 1 | 1 | 8.5 |
| Rebchuk et al., 2021 | 0.5 | 1 | 0.5 | 1 | 0.5 | 1 | 1 | 1 | 0.5 | 7 |
| Samuelsson et al., 2021 | 1 | 1 | 0.5 | 0 | 1 | 1 | 1 | 0.5 | 0.5 | 6.5 |
| Saroja et al., 2020 | 0.5 | 0.5 | 0.5 | 0.5 | 0.5 | 1 | 1 | 1 | 1 | 6.5 |
| Schaapsmeerders et al., 2013 | 1 | 1 | 1 | 1 | 1 | 1 | 1 | 1 | 1 | 9 |
| Si Larbi et al., 2021 | 0.5 | 0.5 | 0.5 | 0 | 0 | 1 | 1 | 1 | 0 | 4.5 |

**Supplementary Table S2.** Overview of the used cognitive measurement tools

| Assessment mode | Test name | Test abbreviation | Cognitive domain | *k* studies | Study |
| --- | --- | --- | --- | --- | --- |
| Global cognition |  |  |  |  |  |
|  | Addenbrooke Cognitive Examination | ACE-III | Global cognition | 2 | Done et al., 2021 Saroja et al., 2020 |
|  | Barrow Neurological Institute Screen for Higher Cerebral Functions | BNIS | Global cognition | 2 | Pedersen et al., 2018 Samuelsson et al., 2021 |
|  | Higher Cortical Function Deficit screening | HCFD | Global cognition | 1 | Hoffmann, 1998 |
|  | Luria's neuropsychological investigation | Luria | Global cognition | 1 | Hoffmann, 1998 |
|  | Mini-Mental State Examination | MMSE | Global cognition | 3 | Cao et al., 2007  Naess et al., 2005 Schaapsmeerders et al., 2013 |
|  | Montreal Cognitive Assessment | MoCA | Global cognition | 4 | Koivunen et al., 2015  Lu et al., 2016  Pinter et al., 2019  Rebchuk et al., 2021 |
|  | National Institutes of Health Toolbox Cognition Battery | NIHTB-CB | Global cognition | 1 | Rebchuk et al., 2021 |
|  | National Institute of Neurological Disorders and Stroke – Canadian Stroke Network 30-min neuropsychological battery | NINDS – CSN VCIHS | Global cognition | 1 | Kapoor et al., 2019 |
|  | Telephone Interview for Cognitive Status - Modified | TICS-m | Global cognition | 1 | Huang et al., 2015 |
|  | South African Wechsler Adult Intelligence Screen | WAIS - South African | Global cognition | 1 | Hoffmann, 1998 |
| Domain Specific |  |  |  |  |  |
|  | Block Designs | BD | Visuospatial reasoning | 1 | Hoffmann, 1998 |
|  | Boston Naming Test | BNT | Language | 2 | Cao et al., 2007  Hoffmann & Cases, 2008 |
|  | Babcock Story Recall Test | BSRT | Episodic Memory | 1 | Cao et al., 2007 |
|  | Color Word Interference test - color naming | CWI-color naming | Processing speed | 1 | Samuelsson et al., 2021 |
|  | Color Word Interference test - inhibition | CWI-inhibition | Processing speed | 1 | Samuelsson et al., 2021 |
|  | Color Word Interference test - inhibition/switching | CWI-inhibition/switching | Executive functioning | 1 | Samuelsson et al., 2021 |
|  | Color Word Interference test - word reading | CWI-word reading | Executive functioning | 1 | Samuelsson et al., 2021 |
|  | Corsi’s block-tapping board | Corsi | Working memory | 1 | Cao et al., 2007 |
|  | Digit Span Test | DST | Working memory | 1 | Hoffmann, 1998 |
|  | Digit Span Test - backward | DST-backward | Working memory | 2 | Cao et al., 2007  Lu et al., 2016 |
|  | Digit Span Test - forward | DST-forward | Working memory | 2 | Cao et al., 2007  Lu et al., 2016 |
|  | Frontal tests battery | FTB | Executive functioning | 1 | Hoffmann & Cases, 2008 |
|  | National Institutes of Health Stroke Scale - item 9 | NIHSS item 9 | Language | 2 | Aarnio et al., 2018  Pinter et al., 2019 |
|  | Norsk Grunntest for Afasi | NGA | Language | 1 | Naess et al., 2009 |
|  | Object Assembly | OA | Spatial visualization | 1 | Hoffmann, 1998 |
|  | Paper and Pencil Memory Scanning Test - DHN | PPMST-DHN | Working memory | 1 | Schaapsmeerders et al., 2013 |
|  | Paper and Pencil Memory Scanning Test - MP | PPMST-MP | Working memory | 1 | Schaapsmeerders et al., 2013 |
|  | Raven’s progressive matrices | RPM | Abstract reasoning | 1 | Cao et al., 2007 |
|  | Rey Auditory Verbal Learning Test | RAVLT | Memory | 1 | Hoffmann, 1998 |
|  | Rey Auditory Verbal Learning Test - delayed | RAVLT-delayed | Delayed memory | 3 | Cao et al., 2007  Lu et al., 2016 Schaapsmeerders et al., 2013 |
|  | Rey Auditory Verbal Learning Test - immediate | RAVLT-immediate | Immediate memory | 3 | Cao et al., 2007  Lu et al., 2016 Schaapsmeerders et al., 2013 |
|  | Rey-Osterrieth Complex Figure | ROCF | Memory | 1 | Hoffmann, 1998 |
|  | Rey-Osterrieth Complex Figure - copy | ROCF-copy | Visuoconstruction | 1 | de Bruijn et al., 2014 |
|  | Rey-Osterrieth Complex Figure - delayed | ROCF-delayed | Delayed memory | 2 | de Bruijn et al., 2014 Schaapsmeerders et al., 2013 |
|  | Rey-Osterrieth Complex Figure - immediate | ROCF-immediate | Immediate memory | 3 | de Bruijn et al., 2014 Hoffmann, 1998 Schaapsmeerders et al., 2013 |
|  | Scandinavian Stroke Scale | SSS | Language | 1 | Naess et al., 2009 |
|  | Similarities | sim | Abstract reasoning | 1 | Cao et al., 2007 |
|  | Star Cancellation test | Star | Processing speed | 1 | Samuelsson et al., 2021 |
|  | Stroop Color-Word Test - I | SCWT-I | Processing speed | 2 | de Bruijn et al., 2014 Schaapsmeerders et al., 2013 |
|  | Stroop Color-Word Test - II | SCWT-II | Processing speed | 2 | de Bruijn et al., 2014 Schaapsmeerders et al., 2013 |
|  | Stroop Color-Word Test - interference | SCWT-interference | Executive functioning (inhibition) | 2 | de Bruijn et al., 2014 Schaapsmeerders et al., 2013 |
|  | Subtest for aphasia | Subtest for aphasia | Language | 1 | Ferro & Crespo, 1988 |
|  | Symbol Digit Modalities Test | SDMT | Processing speed | 5 | de Bruijn et al., 2014 Hoffmann, 1998  Lu et al., 2016  Pinter et al., 2019 Schaapsmeerders et al., 2013 |
|  | Test of Attentional Performance - tonic alertness | TAP-tonic | Processing speed | 1 | Samuelsson et al., 2021 |
|  | Test of Attentional Performance - visuospatial attention | TAP-visuospatial | Processing speed |  | Samuelsson et al., 2021 |
|  | Test of Attentional Performance - phasic attention | TAP-phasic | Processing speed | 1 | Samuelsson et al., 2021 |
|  | Token Test | TT | Language | 2 | Cao et al., 2007  Ferro & Crespo, 1988 |
|  | Trail Making Test | TMT | Processing speed  Executive functioning (switching) | 2 | Hoffmann, 1998  Pinter et al., 2019 |
|  | Trail Making Test A | TMT-A | Executive functioning (swithcing) | 3 | Lu et al., 2016  Pedersen et al., 2018 Samuelsson et al., 2021 |
|  | Trail Making Test B | TMT-B | Processing speed | 2 | Pedersen et al., 2018 Samuelsson et al., 2021 |
|  | Verbal Fluency Test | VF | Language | 1 | Lu et al., 2016 |
|  | Verbal Fluency Test - phonetic | VF-phonetic | Language | 3 | Hoffmann, 1998  Pinter et al., 2019 Samuelsson et al., 2021 |
|  | Verbal Fluency Test - semantic | VF-semantic | Language | 5 | Cao et al., 2007  Hoffmann, 1998  Pinter et al., 2019 Samuelsson et al., 2021 Schaapsmeerders et al., 2013 |
|  | Verbal Series Attention Test | VSAT | Attention | 1 | Schaapsmeerders et al., 2013 |
|  | Wechsler Memory Scale | WMC | Memory | 1 | Hoffmann, 1998 |
|  | Wechsler Memory Scale III - Word Pair test - delayed | WMC-WPT-delayed | Delayed memory | 1 | de Bruijn et al., 2014 |
|  | Wechsler Memory Scale III - Word Pair test - immediate | WMC-WPT-immediate | Immediate memory | 1 | de Bruijn et al., 2014 |
|  | Wisconsin Card Sorting Test | WCST | Executive functioning | 2 | Hoffmann, 1998  Hoffmann & Cases, 2008 |
|  | Zoo Map test | ZOO | Executive functioning | 1 | de Bruijn et al., 2014 |
| Questionnaires |  |  |  | 5 | Dieynabou Sow et al., 2016  Gans et al., 2021  Kim et al., 2005  Mattuzzi & Pfenninger, 2018  Samuelsson et al., 2021 |
| No specific test reported |  |  |  | 6 | Chraa et al., 2014;  Do et al., 2022;  Gonzalez Mc et al., 2017; Lutski et al., 2017;  Moond et al., 2020;  Si Larbi et al., 2021 |

**Supplementary Table S3.** Summary of the studies (*k* = 10) reporting the prevalence of cognitive impairment in young stroke

| Study | Country | Cognitive testing | | Prevalence of cognitive impairment by phase since stroke (%) | |
| --- | --- | --- | --- | --- | --- |
|  |  | Assessment measure | Impairment criteria | not-chronic | chronic |
| Cao et al., 2007 | ITA | **MMSE**  NPS battery | score < 24/30  established cut-off ≥ 3 tests |  | 16 (40%) |
| Done et al., 2021 | IND | **ACE-III** | below 2SD ≥ 2 domains |  | 117 (78%) |
| Gans et al., 2021 | USA | questionnaire | question concentration + memory difficulties | 31 (26%) | |
| Hoffmann, 1998 | ZAF | **HCFD**  NPS battery | according to the norms published by Lezak et al., 2012 | 92 (53%) |  |
| Hoffmann & Cases, 2008 | USA | NPS battery | Nr. | 16 (62%) |  |
| Huang et al., 2015 | CHN | **TICS-m** | score ≤ 31/50 |  | 138 (39%) |
| Naess et al., 2005 | NOR | **MMSE** | score ≤ 25 |  | 52 (27%) |
| Pinter et al., 2019 | AUT | **MoCA**  NPS battery | score < 26  below 1.5 SD ≥ 1 tests | 48 (42%) |  |
| Rebchuk et al., 2021 | CAN | **MoCA**  **NIHTB-CB** | score < 26 | 19 (37%) |  |
| Schaapsmeerders et al., 2013 | NLD | **MMSE**  NPS battery | below 1.5 SD ≥ 1 tests |  | 96 (35%) |

*Notes.* Nr. = Not reported; In **bold**: global cognitive functioning outcome. NPS = Neuropsychological Screening; not-chronic: 0 – 6 months post stroke; chronic > 6 months post stroke;

**Supplementary Table S4.** Summary of the studies (*k* = 13) reporting the prevalence of language impairment in young stroke

| Study (country) | Country | Cognitive testing | | | Prevalence of language impairment by phase since stroke (%) | |
| --- | --- | --- | --- | --- | --- | --- |
|  |  | Measure of impairment | Impairment criteria | not-chronic | | chronic |
| Aarnio et al., 2018 | FIN | NIHSS item 9 | score ≥ 1 | 162 (21%) | |  |
| Chraa et al., 2014 | MAR | Nr. | Nr. | 36 (28%) | |  |
| Dieynabou Sow et al., 2016 | SEN | questionnaire | Nr. | 15 (28%) | |  |
| Do et al., 2022 | TWN | Nr. | Nr. | 214 (3.3%) | |  |
| Ferro & Crespo, 1988 | PRT | aphasia subtest; Token Test | acccording to scores on the aphasia subtest | 166 (65%) | | |
| Gonzalez Mc et al., 2017 | CHL | Nr. | Nr. | 6 (19%) | |  |
| Hoffmann, 1998 | ZAF | Luria's | according to the norms published by Lezak and as per Luria | 35 (20%) | |  |
| Kim et al., 2005 | KOR | questionnaire | Nr. |  | | 15 (16%) |
| Lutski et al., 2017 | ISR | Nr. | Nr. | 116 (35%) | |  |
| Moond et al., 2020 | IND | Nr. | Nr. | 96 (60%) | |  |
| Naess et al., 2009 | NOR | speech subscale of SSS | score < 10 |  | | 20 (10%) |
| Pinter et al., 2019 | AUT | NIHSS item 9 | score ≥ 1 | 22 (19%) | |  |
| Si Larbi et al., 2021 | SAU | Nr. | Nr. |  | | 90 (13%) |

*Notes.* Nr. = Not reported; In **bold**: global cognitive test. NPS = Neuropsychological Screening; not-chronic: 0 – 6 months post stroke; chronic > 6 months post stroke

**Supplementary Table S5.** Summary of the studies (*k* = 6) reporting the severity of impairment for different cognitive domains

| Study | Country | Cognitive domains  (N tests per domain) | Tests |
| --- | --- | --- | --- |
| Cao et al., 2007 | ITA | global cognition (1)  abstract reasoning (2)  delayed memory (1)  immediate memory (2)  language (3)  working memory (3) | **MMSE**  BNT  BSRT  Corsi  DST – backward  DST – forward  RPM  RAVLT – immediate  RAVLT – delayed  Similarities  TT  VF – semantic |
| de Bruijn et al., 2014 | NLD | delayed memory (2)  executive functioning and attention (2)  immediate memory (2)  processing speed (3)  visuoconstruction (1) | ROCF – copy  ROCF – delayed  ROCF – immediate  SCWT – I  SCWT – II  SCWT – interference  SDMT  WMC – WPT – delayed  WMC – WPT – immediate  ZOO |
| Koivunen et al., 2015 | FIN | global cognition (1) | **MoCA** |
| Naess et al., 2005 | NOR | global cognition (1) | **MMSE** |
| Pedersen et al., 2018 | SWE | global cognition (1)  executive functioning and attention (1)  processing speed (1) | **BNIS**  TMT – A  TMT – B |
| Schaapsmeerders et al., 2013 | NLD | global cognition (1)  delayed memory (2)  executive functioning and attention (2)  immediate memory (2)  language (1)  processing speed (3)  visuoconstruction (1)  working memory (2) | **MMSE**  PPMST – DHN  PPMST – MP  RAVLT – delayed  RAVLT – immediate ROCF – copy  ROCF – delayed  ROCF – immediate  SCWT – I  SCWT – II  SCWT – interference  SDMT  VF – semantic  VSAT |

*Notes*. See Table 2 for the full names of the abbreviations of the cognitive tests

**Supplementary Figures**

**Supplementary Figure S1.** Forest plot of the severity of impairment in global cognition


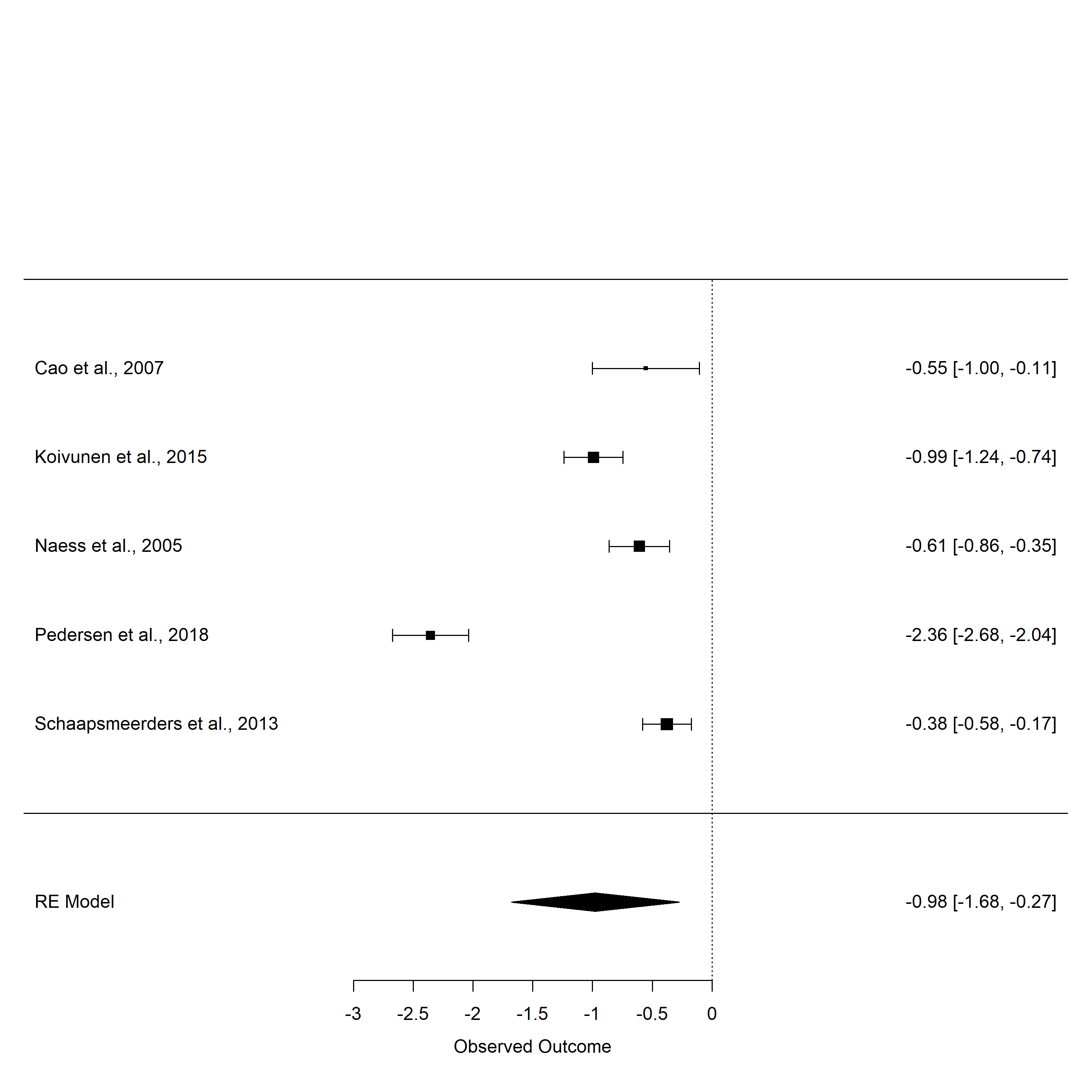


**Supplementary Figure S2**. Forest plot of the severity of impairment in visuoconstruction
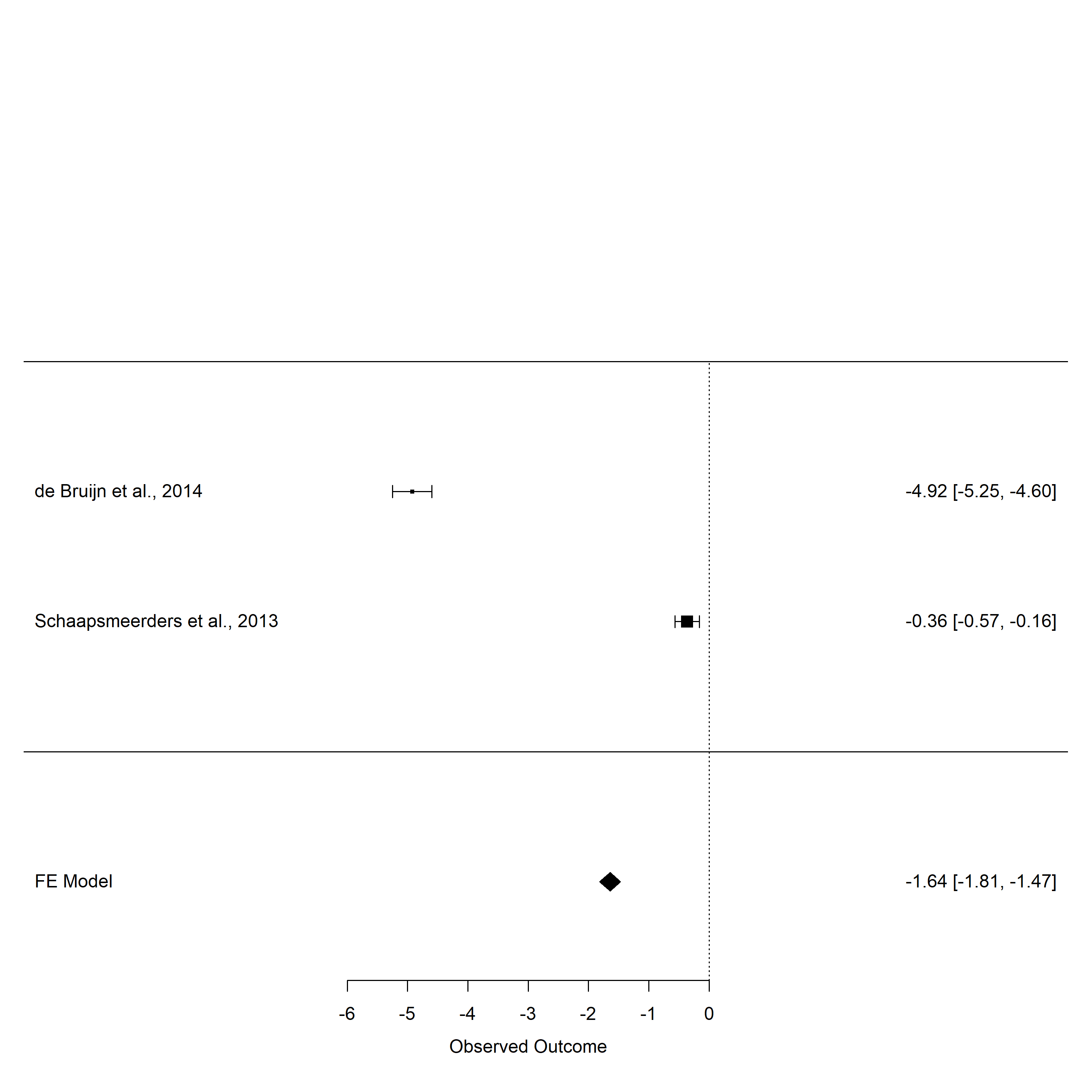


**Supplementary Figure S3.** Forest plot of the severity of impairment in language


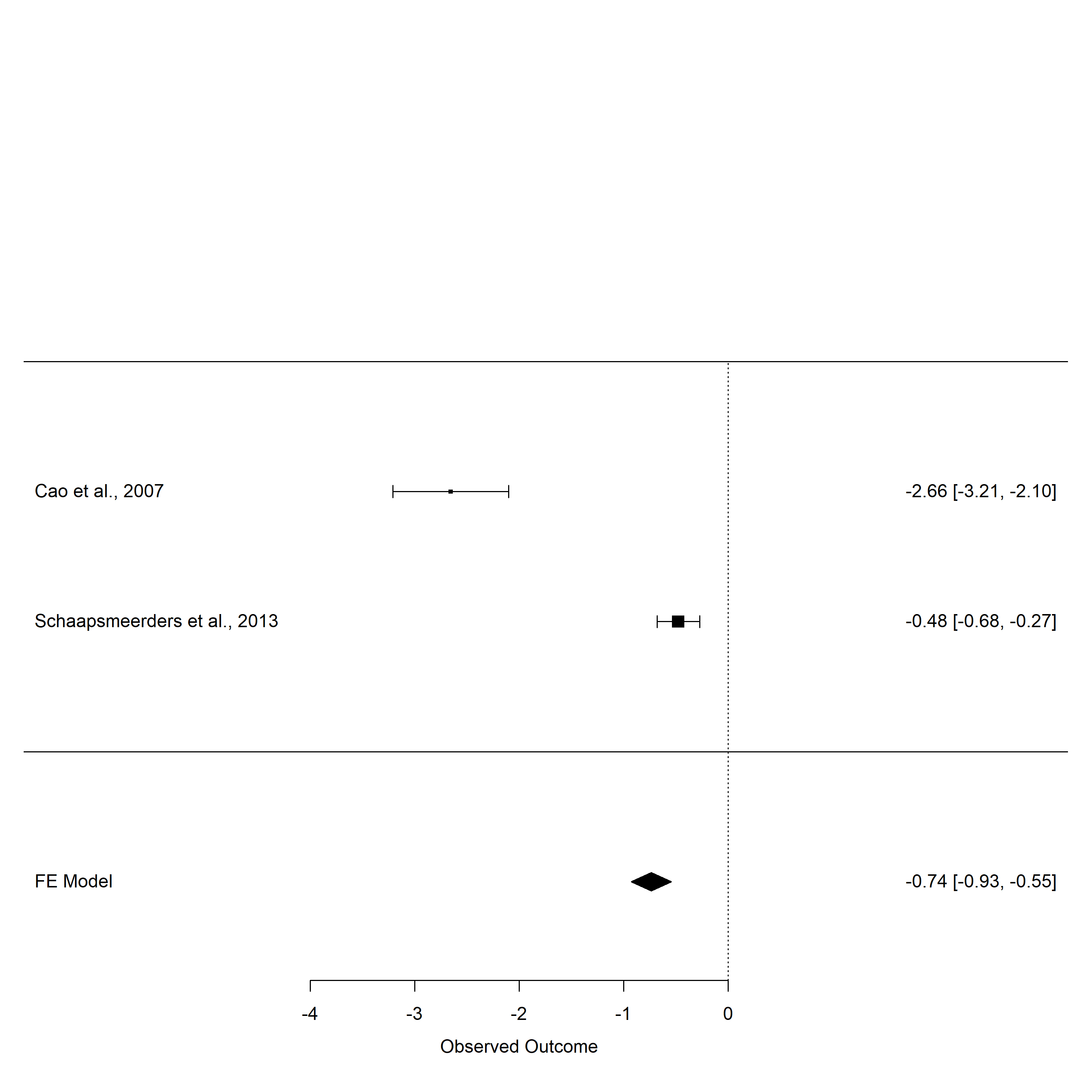


**Supplementary Figure S4.** Forest plot of the severity of impairment in attention and executive functioning


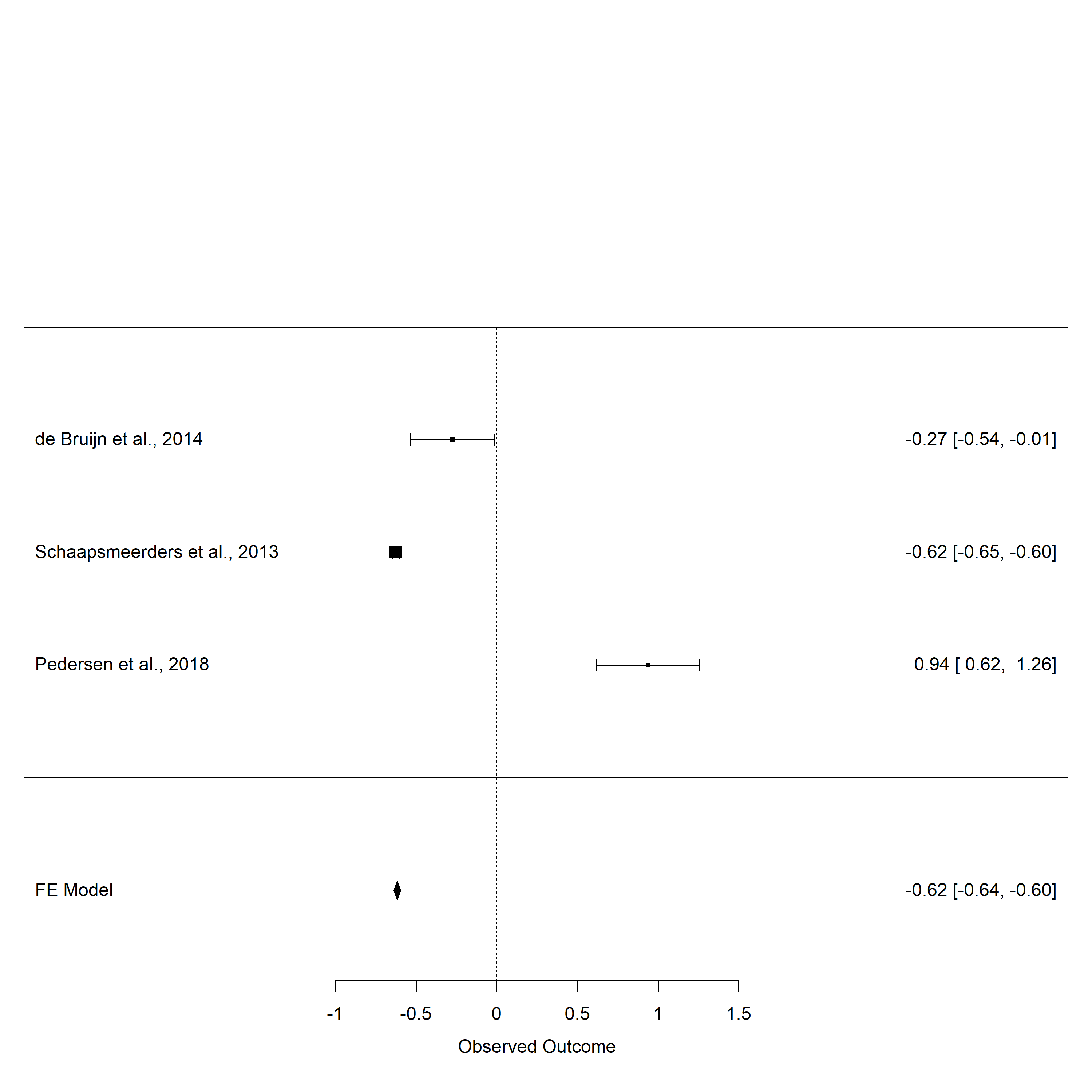


**Supplementary Figure S5.** Forest plot of the severity of impairment in delayed memory


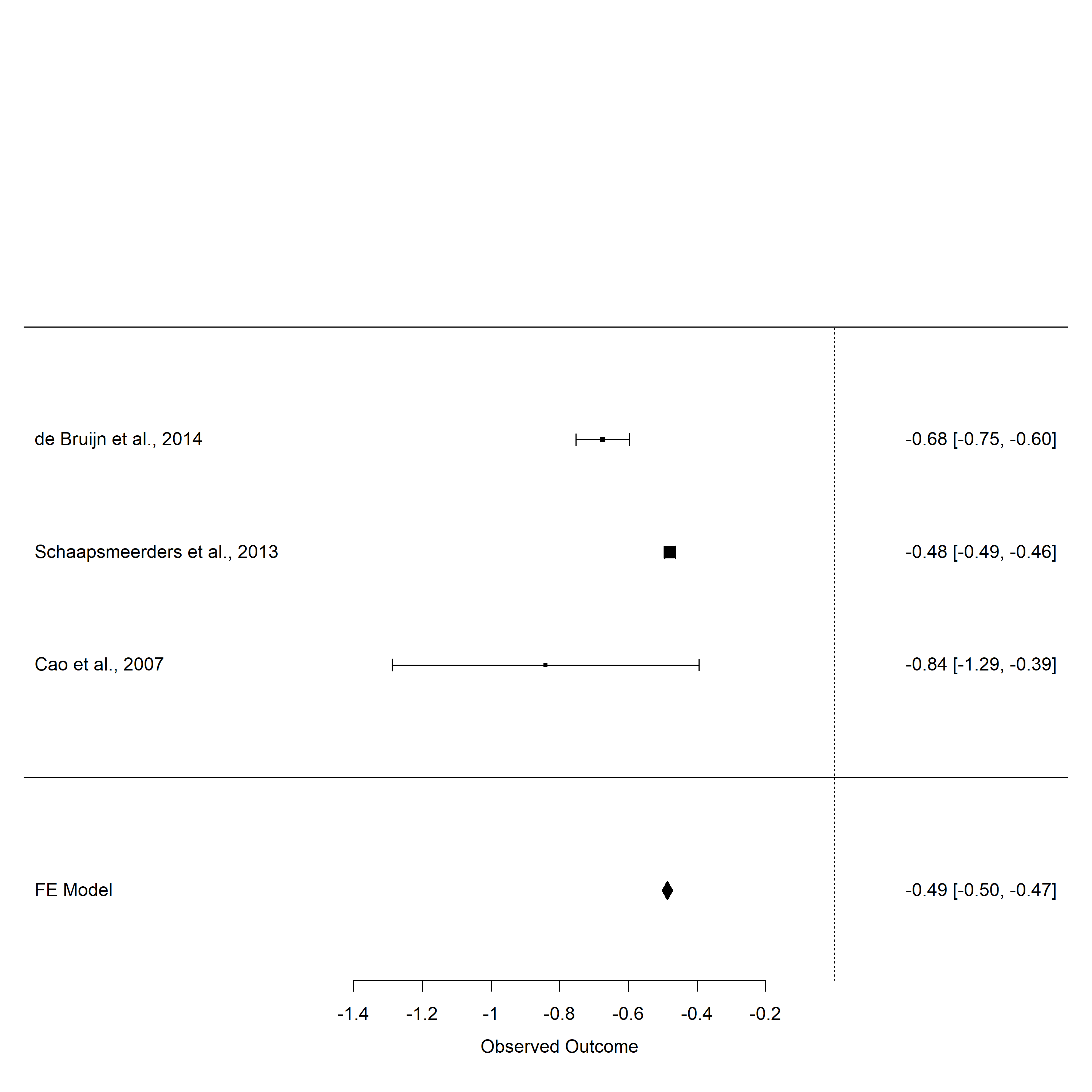


**Supplementary Figure S6.** Forest plot of the severity of impairment in immediate memory
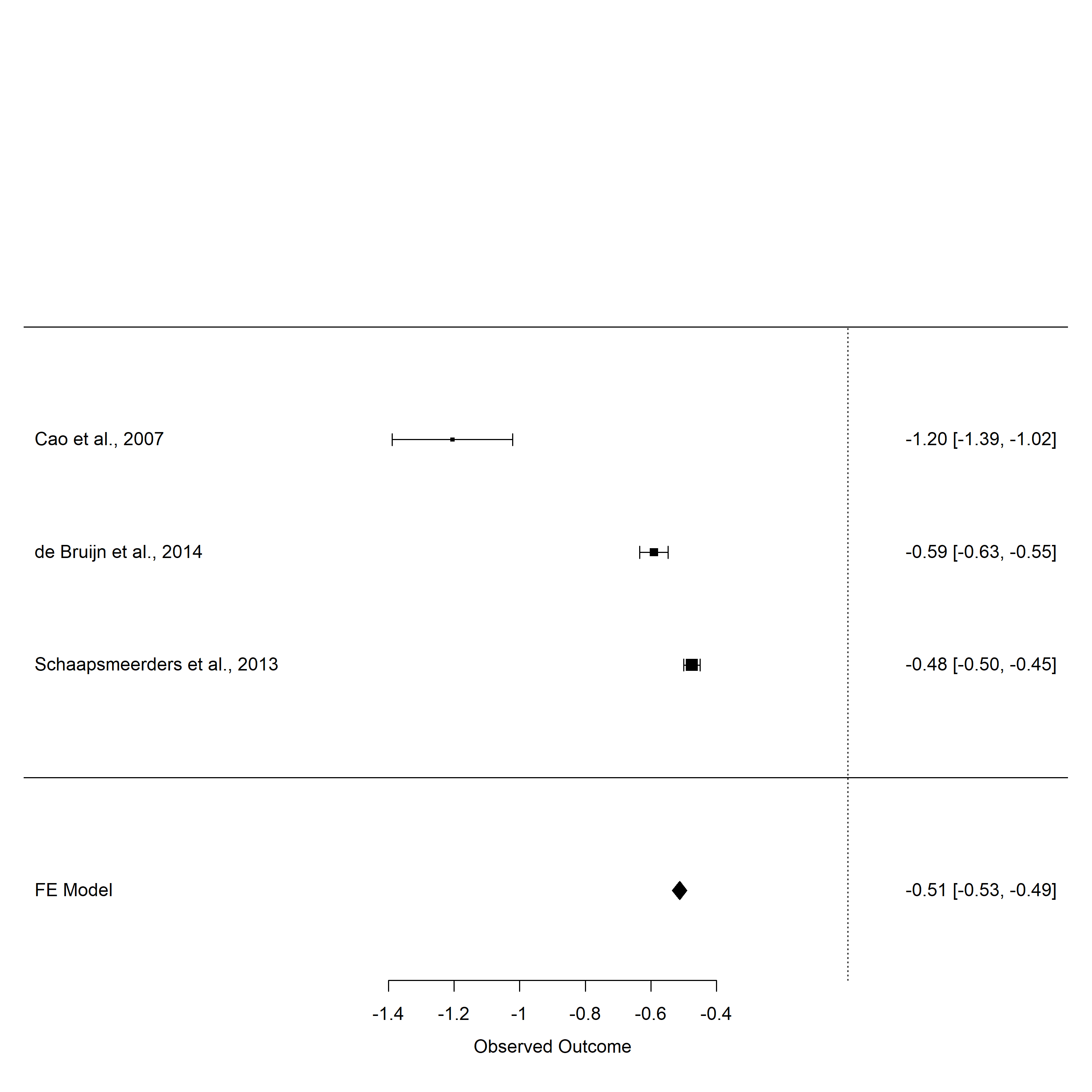


**Supplementary Figure S7.** Forest plot of the severity of impairment in working memory


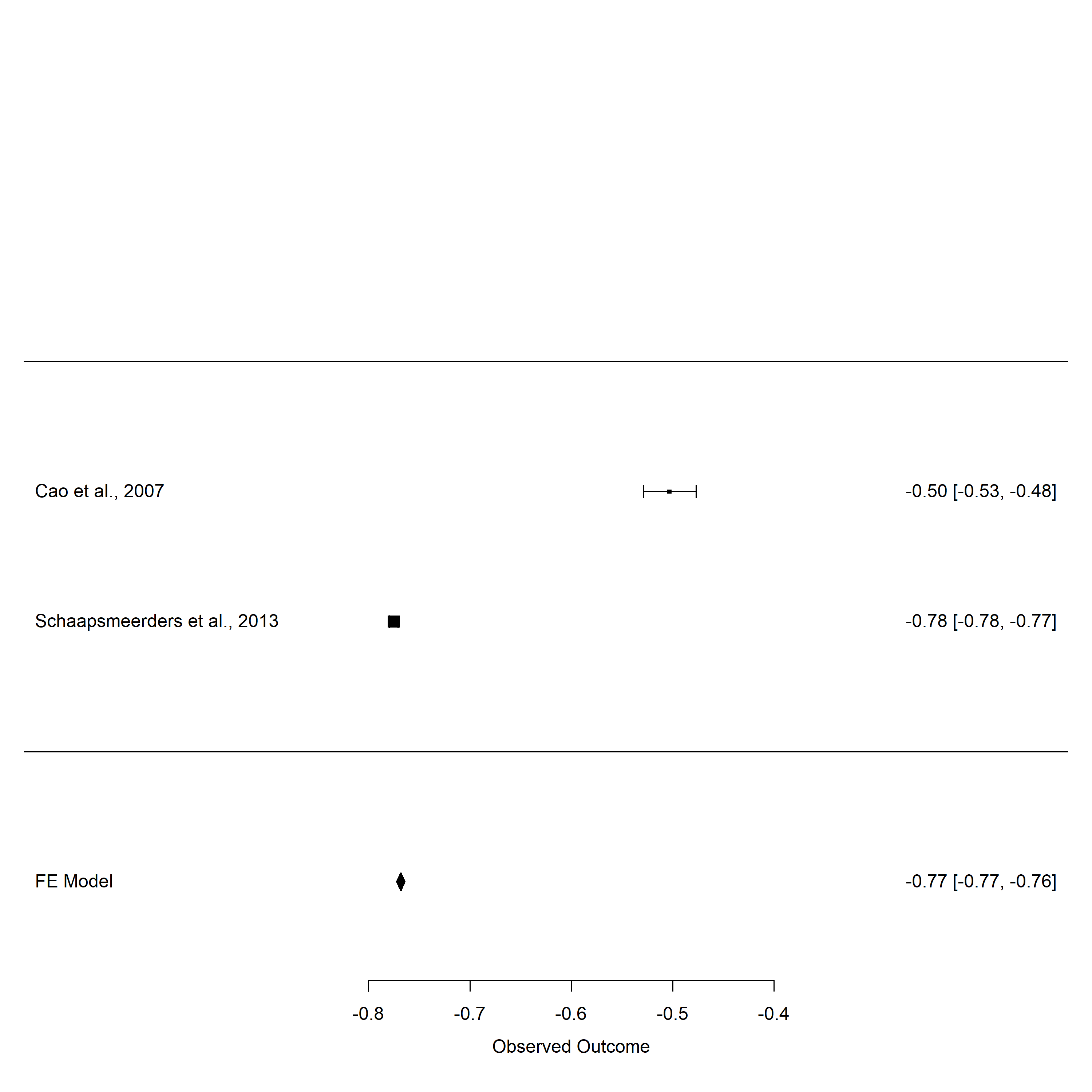


**Supplementary Figure S8.** Forest plot of the severity of impairment in processing speed


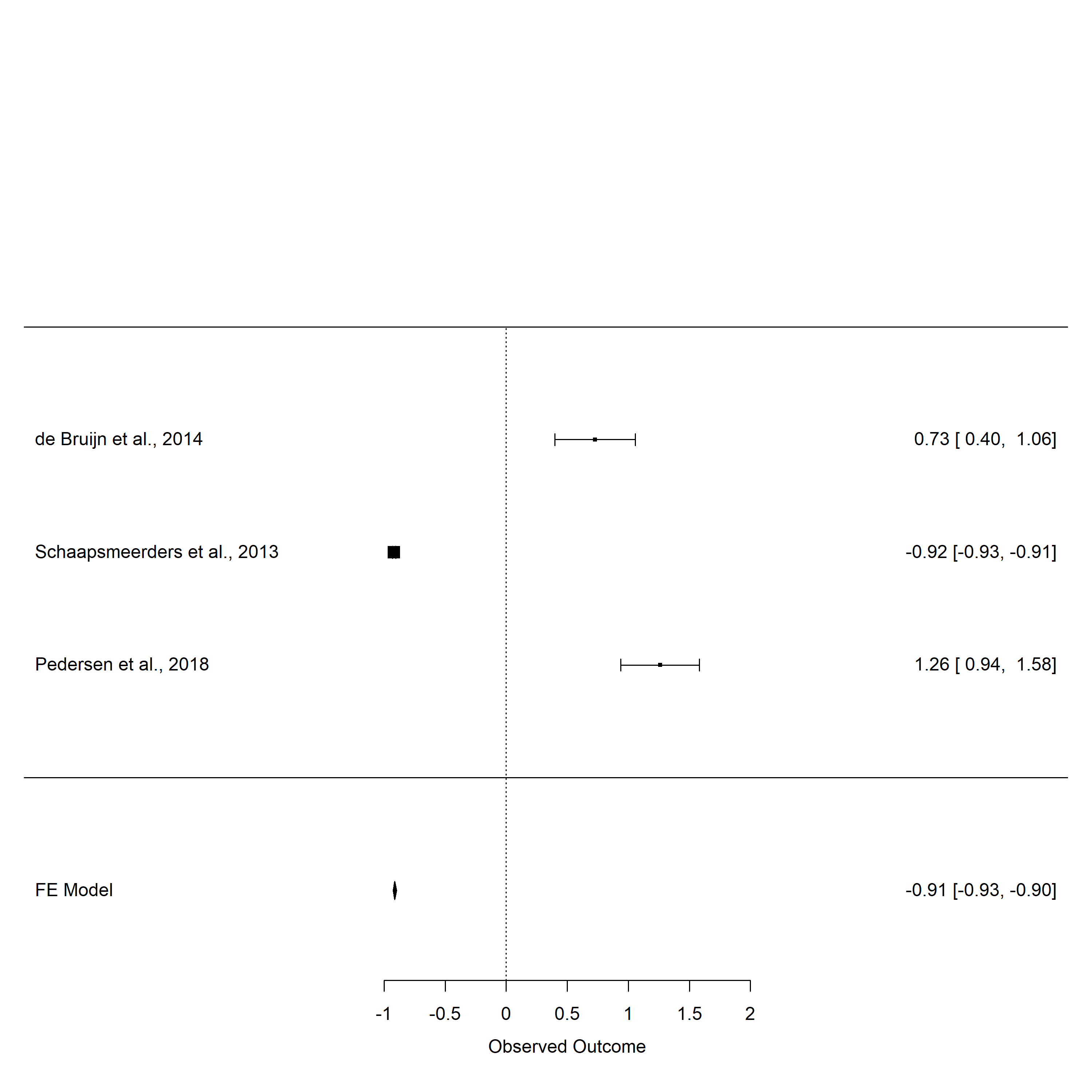


**Reference list for supplementary material**

Aarnio, K., Rodríguez-Pardo, J., Siegerink, B., Hardt, J., Broman, J., Tulkki, L., Haapaniemi, E., Kaste, M., Tatlisumak, T., & Putaala, J. (2018). Return to work after ischemic stroke in young adults: A registry-based follow-up study. *Neurology*, *91*(20), e1909–e1917. https://doi.org/10.1212/WNL.0000000000006510

Cao, M., Ferrari, M., Patella, R., Marra, C., & Rasura, M. (2007). Neuropsychological findings in young-adult stroke patients. *Archives of Clinical Neuropsychology*, *22*(2), 133–142. https://doi.org/10.1016/j.acn.2006.09.005

Chraa, M., Louhab, N., & Kissani, N. (2014). Stroke in young adults: About 128 cases. *The Pan African Medical Journal*, *17*, 37. https://doi.org/10.11604/pamj.2014.17.37.3226

Crowe, M., & Sheppard, L. (2011). A general critical appraisal tool: An evaluation of construct validity. *International Journal of Nursing Studies*, *48*(12), 1505–1516. https://doi.org/10.1016/j.ijnurstu.2011.06.004

de Bruijn, M. A. A. M., Synhaeve, N. E., van Rijsbergen, M. W. A., de Leeuw, F.-E., Jansen, B. P. W., & de Kort, P. L. M. (2014). Long-Term Cognitive Outcome of Ischaemic Stroke in Young Adults. *Cerebrovascular Diseases*, *37*(5), 376–381. https://doi.org/10.1159/000362592

Dieynabou Sow, A., Toure, K., Basse, A. M., & Ndiaye, M. M. (2016). Prognosis of spontaneous hemorrhagic stroke in people under 55 in Senegal, a developing country in Africa: A series of 53 cases. *Pronostic Des Hemorragies Cerebrales Spontanees Du Sujet de Moins de 55 Ans, Dans Un Pays En Developpement d’Afrique de l’Ouest Exemple Du Senegal, a Propos de Cinquante-Trois Cas.*, *26*(2), 170–174. https://doi.org/10.1684/mst.2016.0542

Do, P. T., Chen, L.-Y., Chan, L., Hu, C.-J., & Chien, L.-N. (2022). Risk Factors for Postischemic Stroke Epilepsy in Young Adults: A Nationwide Population-Based Study in Taiwan. *Frontiers in Neurology*, *13*(101546899), 880661. https://doi.org/10.3389/fneur.2022.880661

Done, I. P., Aghoram, R., & Narayan, S. K. (2021). Everyday Abilities Scale for India in Screening for Poststroke Dementia Among Young Stroke Survivors. *Alzheimer Disease and Associated Disorders*, *35*(3), 275–277. https://doi.org/10.1097/WAD.0000000000000452

Ferro, J. M., & Crespo, M. (1988). Young adult stroke: Neuropsychological dysfunction and recovery. *Stroke*, *19*(8), 982–986.

Gans, S. D., Michaels, E., Thaler, D. E., & Leung, L. Y. (2021). Detection of symptoms of late complications after stroke in young survivors with active surveillance versus usual care. *Disability and Rehabilitation*, *9207179, a8i*, 1–5. https://doi.org/10.1080/09638288.2021.1883749

Gonzalez Mc, F., Lavados G, P., & Olavarria I, V. (2017). [Incidence of aphasia in patients experiencing an ischemic stroke]. *Incidencia Poblacional, Caracteristicas Epidemiologicas y Desenlace Funcional de Pacientes Con Ataque Cerebrovascular Isquemico y Afasia.*, *145*(2), 194–200. https://doi.org/10.4067/S0034-98872017000200007

Hoffmann, M. (1998). Stroke in the young: The multiethnic prospective durban stroke data bank results. *Journal of Stroke and Cerebrovascular Diseases : The Official Journal of National Stroke Association*, *7*(6), 404–413.

Hoffmann, M., & Cases, L. B. (2008). Etiology of frontal network syndromes in isolated subtentorial stroke. *Behavioural Neurology*, *20*(3), 101–105. https://doi.org/10.3233/BEN-2008-0220

Huang, Y., Yang, S., & Jia, J. (2015). Factors related to long-term post-stroke cognitive impairment in young adult ischemic stroke. *Medical Science Monitor : International Medical Journal of Experimental and Clinical Research*, *21*(dxw, 9609063), 654–660. https://doi.org/10.12659/MSM.892554

Kapoor, A., Scott, C., Lanctot, K. L., Herrmann, N., Murray, B. J., Thorpe, K. E., Lien, K., Sicard, M., & Swartz, R. H. (2019). Symptoms of depression and cognitive impairment in young adults after stroke/transient ischemic attack. *Psychiatry Research*, *279*(qc4, 7911385), 361–363. https://doi.org/10.1016/j.psychres.2019.06.022

Kim, J. S., Choi-Kwon, S., Kwon, S. U., Lee, H. J., Park, K.-A., & Seo, Y. S. (2005). Factors affecting the quality of life after ischemic stroke: Young versus old patients. *Journal of Clinical Neurology (Seoul, Korea)*, *1*(1), 59–68. https://doi.org/10.3988/jcn.2005.1.1.59

Koivunen, R.-J., Harno, H., Tatlisumak, T., & Putaala, J. (2015). Depression, anxiety, and cognitive functioning after intracerebral hemorrhage. *Acta Neurologica Scandinavica*, *132*(3), 179–184. https://doi.org/10.1111/ane.12367

Lezak, M. D., Howieson, D. B., Bigler, E. D., & Tranel, D. (2012). *Neuropsychological Assessment*. OUP USA.

Lu, D., Ren, S., Zhang, J., & Sun, D. (2016). Vascular risk factors aggravate cognitive impairment in first-ever young ischaemic stroke patients. *European Journal of Neurology*, *23*(5), 940–947. https://doi.org/10.1111/ene.12967

Lutski, M., Zucker, I., Shohat, T., & Tanne, D. (2017). Characteristics and outcomes of young patients with first-ever ischemic stroke compared to older patients: The national acute stroke Israeli registry. *Frontiers in Neurology*, *8*(AUG), 421. https://doi.org/10.3389/fneur.2017.00421

Mattuzzi, M., & Pfenninger, S. E. (2018). The language-cognition-affect interface in young college student stroke survivors with aphasia. *International Journal of Applied Linguistics*, *28*(3), 465–479. https://doi.org/10.1111/ijal.12222

Moond, V., Bansal, K., & Jain, R. (2020). Risk Factors and Subtyping of Ischemic Stroke in Young Adults in the Indian Population. *Cureus*, *12*(11), e11388. https://doi.org/10.7759/cureus.11388

Naess, H., Hammersvik, L., & Skeie, G. O. (2009). Aphasia among Young Patients with Ischemic Stroke on Long-term Follow-up. *Journal of Stroke and Cerebrovascular Diseases*, *18*(4), 247–250. https://doi.org/10.1016/j.jstrokecerebrovasdis.2008.10.005

Naess, H., Nyland, H. I., Thomassen, L., Aarseth, J., & Myhr, K.-M. (2005). Mild depression in young adults with cerebral infarction at long-term follow-up: A population-based study. *European Journal of Neurology*, *12*(3), 194–198. https://doi.org/10.1111/j.1468-1331.2004.00937.x

Pedersen, A., Stanne, T. M., Redfors, P., Viken, J., Samuelsson, H., Nilsson, S., Jood, K., & Jern, C. (2018). Fibrinogen concentrations predict long-term cognitive outcome in young ischemic stroke patients. *Research and Practice in Thrombosis and Haemostasis*, *2*(2), 339–346. https://doi.org/10.1002/rth2.12078

Pinter, D., Enzinger, C., Gattringer, T., Eppinger, S., Niederkorn, K., Horner, S., Fandler, S., Kneihsl, M., Krenn, K., Bachmaier, G., & Fazekas, F. (2019). Prevalence and short-term changes of cognitive dysfunction in young ischaemic stroke patients. *European Journal of Neurology*, *26*(5), 727–732. https://doi.org/10.1111/ene.13879

Rebchuk, A. D., Kuzmuk, L. E., Deptuck, H. M., Silverberg, N. D., & Field, T. S. (2021). Evaluating High-Functioning Young Stroke Survivors with Cognitive Complaints. *Canadian Journal of Neurological Sciences*, 1–5. https://doi.org/10.1017/cjn.2021.137

Samuelsson, H., Viken, J., Redfors, P., Holmegaard, L., Blomstrand, C., Jern, C., & Jood, K. (2021). Cognitive function is an important determinant of employment amongst young ischaemic stroke survivors with good physical recovery. *European Journal of Neurology*, *28*(11), 3692–3701. https://doi.org/10.1111/ene.15014

Saroja, A. O., Thorat, N. N., & Naik, K. R. (2020). Depression and Quality of Life after Cerebral Venous Sinus Thrombosis. *Annals of Indian Academy of Neurology*, *23*(4), 487–490. https://doi.org/10.4103/aian.AIAN_191_19

Schaapsmeerders, P., Maaijwee, N. A. M., van Dijk, E. J., Rutten-Jacobs, L. C. A., Arntz, R. M., Schoonderwaldt, H. C., Dorresteijn, L. D. A., Kessels, R. P. C., & de Leeuw, F.-E. (2013). Long-term cognitive impairment after first-ever ischemic stroke in young adults. *Stroke*, *44*(6), 1621–1628. https://doi.org/10.1161/STROKEAHA.111.000792

Sexton, E., McLoughlin, A., Williams, D. J., Merriman, N. A., Donnelly, N., Rohde, D., Hickey, A., Wren, M.-A., & Bennett, K. (2019). Systematic review and meta-analysis of the prevalence of cognitive impairment no dementia in the first year post-stroke. *European Stroke Journal*, *4*(2), 160–171. https://doi.org/10.1177/2396987318825484

Si Larbi, M. T., Al Mangour, W., Saba, I., Al Naqeb, D., Faisal, Z. S., Omar, S., & Ibrahim, F. (2021). Ischemic and Non-ischemic Stroke in Young Adults—A Look at Risk Factors and Outcome in a Developing Country. *Cureus*, *13*(8), e17079. https://doi.org/10.7759/cureus.17079
